# _102_No^2+^ as a soft metal ion: Extraction of ^255^No using crown ethers

**DOI:** 10.1126/sciadv.aeh4903

**Published:** 2026-09-30

**Authors:** Eisuke Watanabe, Ruilin Wang, Takuya Yokokita, Ryohei Nakanishi, Saki Otaka, Ryotaro Masuda, Yudai Itakura, Yudai Shigekawa, Hiromitsu Haba, Akihiro Nambu, Xiaojie Yin, Kyosuke Shibamoto, Masashi Kaneko, Koichi Takamiya, Atsushi Shinohara, Yoshitaka Kasamatsu

**Affiliations:** ^1^ Graduate School of Science, the University of Osaka, Machikaneyamacho 1-1, Toyonaka, Osaka 560-0043, Japan.; ^2^ Nishina Center for Accelerator-based Science, RIKEN, Hirosawa 2-1, Wako, Saitama 351-0198, Japan.; ^3^ High Energy Accelerator research Organization (KEK), Oho 1-1, Tsukuba, Ibaraki, 305-0801 Japan.; ^4^ Research Center for Accelerator and Radioisotope Science, Tohoku University.; ^5^ Institute for Integrated Radiation and Nuclear Science, Kyoto University, Asashiro-Nishi 2, Kumatori, Osaka 590-0494 Japan.; ^6^ Institute for Radiation Sciences, the University of Osaka, Yamadaoka 2-4, Suita 565-0871, Japan.; ^7^ Osaka Aoyama University, Niina 2-11-1, Minoh, Osaka 562-8580, Japan.

## Abstract

The chemical properties of transfermium elements with atomic numbers >100 are largely unknown. Element 102, nobelium, is an actinide and forms stable +2 valent cations in aqueous solutions, unlike other heavy actinide. In previous experiments, No^2+^ exhibits similar behavior to alkaline earth metal ions, particularly Ca^2+^ and Sr^2+^, suggesting hard-acid character. In this study, the soft metal ion behavior of No^2+^ was demonstrated through complex with crown ethers. In the solid–liquid extraction of ^255^No using di-^t^Bu-dicyclohexano-18-crown-6 in HNO_3_, No^2+^ was extracted as much as Sr^2+^. Density functional theory calculations confirmed higher stability of the No^2+^–DtBuCH18C6 complex, with covalent bonding involving the 6*d* orbitals. Theoretical studies also revealed strong relativistic effects on the atomic orbitals of No^2+^, rendering No^2+^ a soft ion similar to Pb^2+^.

## INTRODUCTION

The unique chemical properties of transfermium elements with atomic numbers *Z* > 100 can be attributed to the significant relativistic effects on their valence orbitals (*1*, *2*). However, the relativistic effects on atoms remain poorly understood; consequently, the results of detailed chemical experiments using these elements are of great importance. Transfermium elements are radioactive elements that can only be synthesized by heavy-ion-induced nuclear reactions using an accelerator. Because transfermium elements are typically produced in very low yields and many of their experimentally accessible isotopes have short half-lives (*3*), their chemical properties are often investigated using atom-at-a-time techniques (*1*). Therefore, the execution of chemical experiments on these elements is challenging, and their chemical properties remain generally unknown. In the context of liquid-phase chemistry, two-phase partitioning experiments, such as solid–liquid and liquid–liquid extraction, have been conducted, and complexation with inorganic ions or chelating ligands in solution has been discussed based on existing ratios between two phases (*4*, *5*).

Element 102, nobelium, is the second-to-last element in the actinide series (*6*). Early studies performed in the 1960s and 1970s showed that No preferentially forms No^2+^ rather than No^3+^ in aqueous solutions, although other heavy actinides and all lanthanides show preference for the +3 state (*7*). Furthermore, solvent extraction experiments have revealed that No^2+^ behaves more like an s-block element (Ca, Sr, or Ba) than a d-block element (*8*). In elution experiments performed using a cation-exchange column in a bis(2-ethylhexyl) phosphoric acid (HDEHP)/HCl system, the No fraction was eluted from the column at a column volume between those of Ca and Sr (*8*). Additionally, it has been demonstrated that the tendency for No complexation with citrate, oxalate, and acetate ions during solvent extraction in NH_4_NO_3_ fell between those of Ca and Sr (*9*). Notably, the order of extraction for these elements is consistent with the order of their ionic radii (*8*), suggesting that No^2+^ exhibits ionic behavior similar to those of hard ions, such as Ca^2+^ and Sr^2+^ (*10*). Furthermore, Toyoshima *et al.* demonstrated the high stability of divalent No cations in aqueous solutions using flow electrolytic column chromatography (*11*).

Nobelium has been considered to have similar chemical properties to Ca and Sr for the last half-century, suggesting a strong contraction of its fully occupied f orbitals. Yb possesses the same s^2^f^14^ electronic configuration as No in the neutral atomic state. Although oxidation of Yb^2+^ to Yb^3+^ involves opening of the closed 4f^14^ shell, Yb^3+^ is thermodynamically more stable than Yb^2+^ in aqueous solution (*12*–*14*). In contrast, No preferentially exists in the divalent state in aqueous solution, and this behavior may be influenced by strong relativistic effects. Additionally, previous solvent extraction experiments performed using trioctylamine, which concluded that the behavior of No differs from those of Cd and Hg, focused only on the tendency to form tetrachloride ionic complexes (MCl_4_^2−^) (*8*). Nevertheless, chemical studies on No are limited and its chemical properties remain largely unknown. Indeed, previous studies have largely focused only on the adsorption and extraction behaviors of No in solution chemistry, and neither its coordination structures nor the electronic states of its chemical species have been discussed. Very recently, it was reported that No^2+^ stably forms [No(H_2_O)*_n_*]^2+^ (*n* = 1–5) and [No(N_2_)_m_]^2+^ (*m* = 1–3) with 7 s and 6d orbitals of No in the gas phase (*15*). It is therefore desirable to verify complexation behavior of No in solution, where various compounds can be investigated. Through atom-at-a-time experiments and interpretation by theoretical chemistry, the chemical properties of nobelium would be understood in the viewpoint of the relativistic effects on electronic orbitals.

When performing theoretical studies, it is essential to construct a sufficient molecular model. For single atoms of heavy elements, the structure of the chemical species cannot be experimentally identified, and theoretical investigations have been limited to simple interactions with inorganic ions. Thus, to clarify the extracted chemical species, a unique extraction system based on the use of chelating agents to encapsulate the metal ions may be useful. In this context, crown ethers have the potential to satisfy this requirement and represent a promising candidate for heavy metal ion extraction (*16*–*18*). Although crown ethers are typically employed in liquid-liquid extraction (*19*–*23*), we adopted them in a solid–liquid extraction with crown-ether-based resin expecting rapid reaching of extraction reactions to equilibrium state and rapid preparation of dried samples for α-particle measurement (*24*). Furthermore, Sr resin, which contains di-^t^Bu-dicyclohexano-18-crown-6 (DtBuCH18C6), has been widely used for Sr separation in which the structure of the Sr complex is identified (*25*, *26*). Numerous studies have also focused on the development and application of this resin (*27*–*32*).

In this study, the extraction of ^255^No was performed using DtBuCH18C6 to understand interactions between No^2+^ and the crown ether. An online solid–liquid extraction experiment was performed for ^255^No using Sr resin using an automated batch-type extraction apparatus (AMBER) that was previously constructed by our group (*33*, *34*). Additionally, based on the results of density functional theory (DFT) calculations, the electronic state of the No^2+^–crown ether complex is calculated, and the extraction behavior is discussed. Furthermore, No^2+^ is compared with other divalent metal ions to clarify its soft acidic properties.

## RESULTS

### Solid–liquid extraction of ^255^No

Prior to performing the online experiment for ^255^No, offline experiments were conducted using ^47^Ca, ^85^Sr, ^133^Ba, and ^226^Ra radiotracers to determine suitable extraction conditions. To obtain the equilibrium distribution coefficient (*K*_d_) of ^255^No (*T*_1/2_ = 210 s), the extraction reactions must rapidly reach reaction equilibrium. The dependence of the *K*_d_ values of group 2 elements on the mixing time indicated that the solid–liquid extraction reactions in the Sr resin/HNO_3_ and Sr resin/HCl systems reached chemical equilibrium within 180 s of mixing (fig. S9). The obtained *K*_d_ values under chemical equilibrium agree with literature values (*27*), indicating that the developed conditions were suitable for the chemical study of ^255^No. In addition, the *K*_d_ values of ^133^Ba obtained using AMBER (offline conditions) were in good agreement with those obtained by the batch experiment (see Supplementary Materials).

Figure 1 shows the experimental setup used for the online solid–liquid extraction of ^255^No. The ^255^No nuclide was produced by the ^248^Cm(^12^C, 5n) reaction (*35*) using an azimuthally varying-field (AVF) cyclotron at the RIKEN RI Beam Factory (Wako city, Japan). The ^12^C beam was extracted with an energy of 89.9 MeV at ∼0.7 particle-μA. The 685.2 μg/cm^2^ of ^248^Cm and 69.6 μg/cm^2^ of ^nat^Gd mixture on the 1.880 mg/cm^2^ of Be backing foil was used as the target. The nuclear reaction products absorbed on KCl aerosols were transported to a chemistry laboratory by He gas at a flow rate of 2.5 L/min. Following the dissolution of ^255^No in 3.5 M HNO_3_ or 7.6 M HCl solution, Sr resin was brought into contact with the aqueous phase for 60–180 s in the reaction container of the AMBER system. Simultaneously, the ^162^Yb (*T*_1/2_ = 18.9 min) nuclides were produced by the ^nat^Gd(^12^C,x*n*)^162^Yb reaction and the radioactivity of the transported ^162^Yb was monitored during a 1-d online experiment. The transported radioactivity from the accelerator during the online experiments was stable. The chemical yields were defined based on the dissolution efficiency, adsorption to the resin (in the case of extraction with the Sr resin), and efficiency of evaporation to dryness. It should be noted that, when evaluating the *K*_d_ values of Er and No, the chemical yield obtained for ^162^Yb during the solid–liquid extraction was corrected on the basis of 5% Yb extraction using ∼30 mg of the Sr resin, as reported by Filosofov *et al.* (*27*). The chemical yield under each experimental condition remained stable during the online experiment. In the effective 72 cycles of the repeated extraction experiments, 173 α events from ^255^No were registered as listed in table S2. In the present study, the estimated half-life of ^255^No was 212 ± 24 s, which is in good agreement with the reported value of 211 ± 11 s by Asai *et al.* (*36*). The obtained *K*_d_ values for Er in 3.5 M HNO_3_, determined for the first time in this extraction system, ranged from 0.5 to 5 mL g^−1^, consistent with the possible values for f-block elements as estimated from the values reported for Yb and Ac in 4 M HNO_3_ (*27*).

**Fig. 1.**
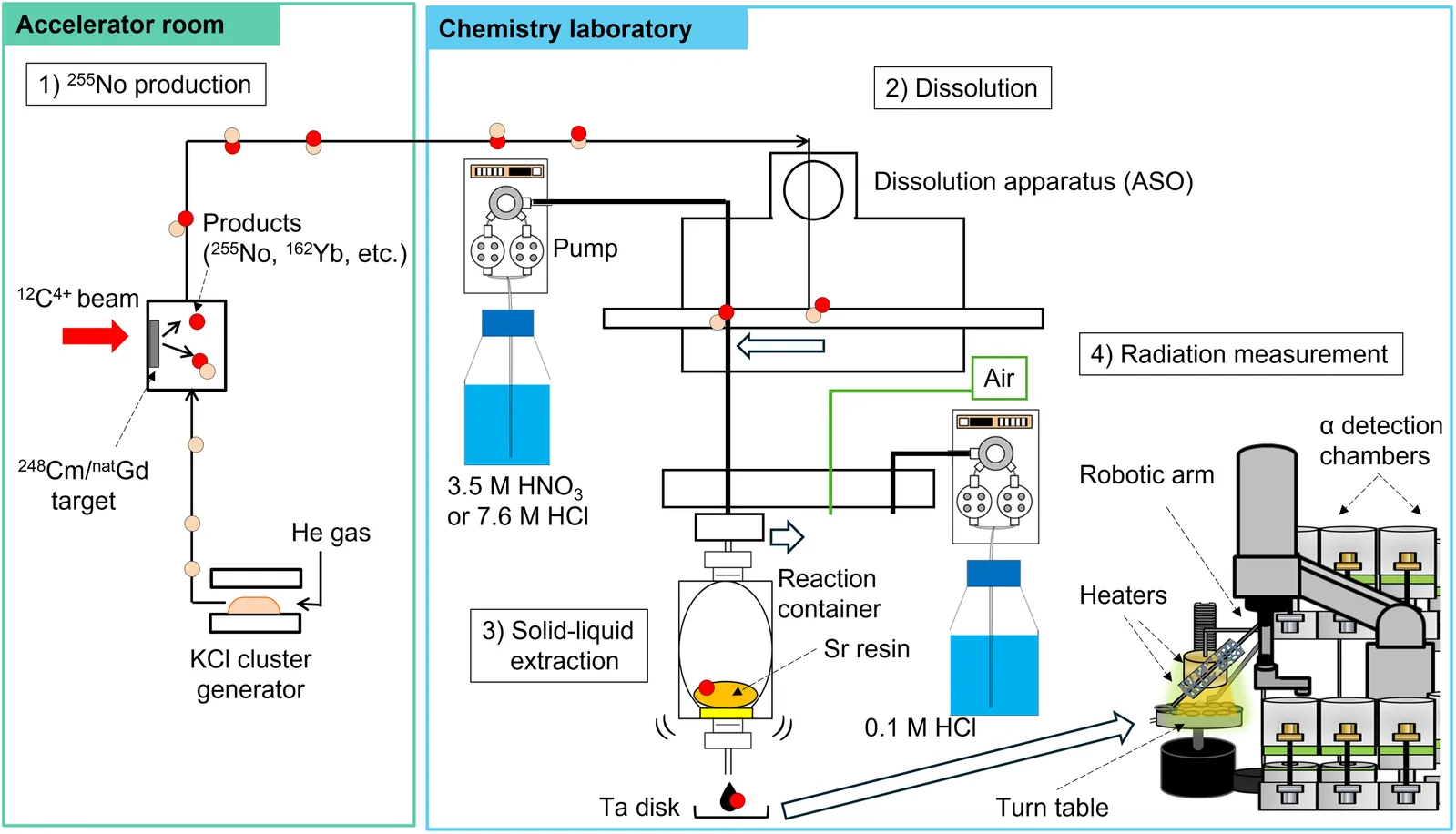
Schematic representation of the online solid–liquid extraction of ^255^No at RIKEN. (1) In the accelerator room, the nuclear reaction products recoil from the target and are caught by KCl aerosol clusters. The products, including ^255^No atoms, are rapidly transferred to a chemistry laboratory by the He/KCl gas-jet system. (2) In the chemistry laboratory, the products are dissolved in 3.5 M HNO_3_ or 7.6 M HCl using dissolution apparatus (ASO). (3) The solution is injected into the reaction container of AMBER and brought into contact with the Sr resin for 60–180 sec. Subsequently, compressed air is used to eject the aqueous phase out onto a Ta dish. The resin remaining in the reaction container is washed with 0.1 M HCl prior to the subsequent solid–liquid extraction. (4) The aqueous sample on the Ta disk is subsequently dried and is finally subjected to α-particle and γ-ray measurements. Illustrations of several components of the experimental apparatus were reproduced from (*5*).

Figure S3 shows the dependence of the *K*_d_ value of No in 3.5 M HNO_3_ on the mixing time in the extraction with the Sr resin. The *K*_d_ value of No appeared to increase upon prolonging the mixing time from 60 to 180 s, although the errors were large. The *K*_d_ value of No at 180 s was comparable to or slightly higher than the *K*_d_ value of Sr under equilibrium. Because the extraction reaction was expected to proceed further with increasing contact time with the resin, the *K*_d_ value obtained at 180 s was used as a tentative representative value in this study. The order of the ionic radii is assumed to be Ca^2+^ < No^2+^ < Sr^2+^ (*8*), and the inclusion selectivity for the crown ether was also considered to follow this order. However, the strong reactivity of No^2+^, which cannot be explained only by the ionic radius, indicates that No^2+^ exhibits a distinctly different reactivity with the crown ether compared to Ca^2+^ and Sr^2+^. In the Sr resin/HCl extraction system, the *K*_d_ value of No was obtained at a mixing time of 60 s and is comparable to the low *K*_d_ values of Sr, Ba, and Ra (fig. S4). In the HCl system, Sr^2+^ is extracted as a Sr[DCH18C6(H_2_O)_2_]^2+^ species, as revealed using extended X-ray absorption fine structure (EXAFS) analysis (*25*). It was therefore expected that all divalent metal ions including No^2+^ may be extracted in the form of M[DtBuCH18C6(H_2_O)_2_]^2+^ (M = Ca, Sr, Ba, Ra, and No).

In this study, comparative elements were employed to consider the possibility that the complexation behavior of No^2+^ deviates from the simple tendency of the ionic radius among alkaline earth metals (hard acids). Previously, Horwitz *et al.* obtained *K*_d_ values for the extraction of radiotracers for alkali metal, and alkaline earth metals, actinide ions, Tc, Po, and Pb using Sr resin (*24*). They reported that Pb^2+^ was absorbed more strongly than Sr^2+^ on the Sr resin in HNO_3_. The ionic radius of Pb^2+^ (1.19 Å at coordination number (CN) = 6) (*37*) is similar to those of No^2+^ (1.0–1.1 Å at CN = 6) (*8*) and Sr^2+^ (1.13 Å at CN = 6) (*38*), and the crystal structure of Pb(NO_3_)_2_(18-crown-6) is similar to that of its Sr analog (*39*). It was therefore assumed that Pb^2+^ was extracted into the Sr resin with a similar coordination structure to that of Pb(NO_3_)_2_(DtBuCH18C6).

Figure S9 shows the time dependence of the *K*_d_ values for ^212^Pb, indicating that the adsorption kinetics of Pb^2+^ are relatively slow in reaching chemical equilibrium. This behavior of Pb^2+^ differs from those exhibited by alkaline earth element ions, but may be similar to that of No^2+^. Nevertheless, it appears that the chemical reaction of Pb^2+^ almost reached equilibrium within 3 min. Unfortunately, it was not possible to obtain the *K*_d_ value of ^255^No for mixing times >3 min due to restrictions associated with the extraction apparatus. Based on the fact that equilibrium was reached within 3 min for a wide variety of divalent metal ions including soft acid ions and Pb^2+^, we judged that a reaction system with ^255^No could also reach extraction equilibrium within 3 min, taking into account the large errors.

Figure 2 plots the distribution coefficients of No, Ca, Sr, Ba, Ra, and Pb against the ionic radius. The distribution coefficient of No is slightly higher than the values indicated by the fitted curves for Ca, Sr, Ba, and Ra. To determine whether the extraction behavior can be explained by ionic size matching, we constructed a size-based reference trend using the *K*_d_ values of the alkaline-earth metal ions Ca^2+^, Sr^2+^, Ba^2+^, and Ra^2+^. Although the ionic radius of No^2+^ has not precisely established, a representative ionic radius of 1.05 Å, corresponding to the midpoint of the reported range of 1.0–1.1 Å, was adopted (*8*) . The *K*_d_ value of No^2+^ obtained after 180-s shaking lay above the quadratic reference trend, with a deviation exceeding 3σ_emp_. This result suggests that the extraction behavior of No^2+^ cannot be fully described by ionic size alone. Detailed analyses of the deviation bands and the *K*_d_ deviation from the reference trend over the possible No^2+^ ionic-radius range of 1.0–1.1 Å are provided in the Supplementary Materials.

**Fig. 2.**
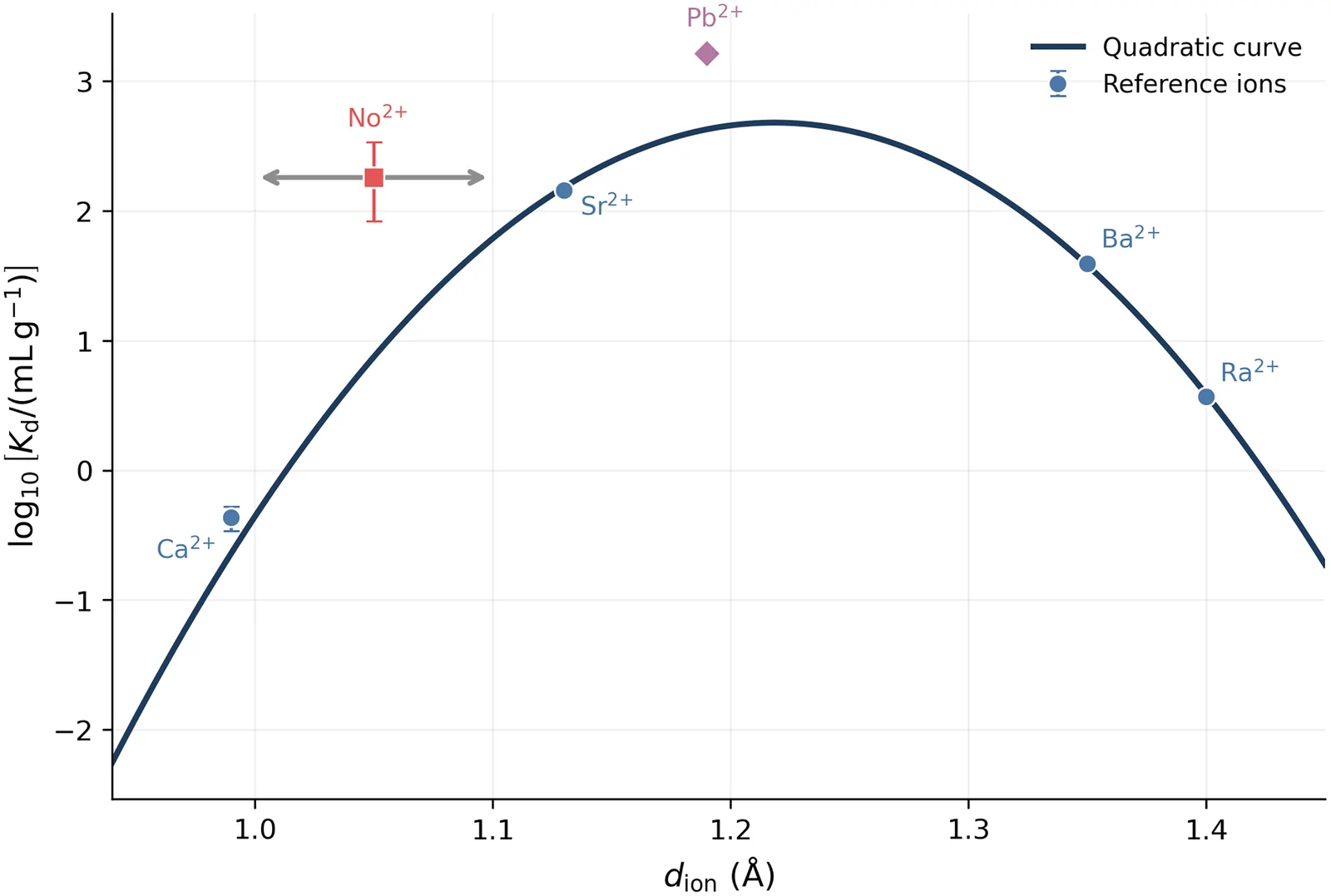
*K*_d_ values of Ca, Sr, Ba, Ra, Pb, and No with the Sr resin in a 3.5 M HNO_3_ solution. The *K*_d_ value for No was that obtained for 180-s shaking. The horizontal double-headed arrow indicates the reported ionic-radius range of 1.00–1.10 Å for No^2+^, and the plotted No^2+^ point corresponds to the representative value of 1.05 Å. The error bars for Ca^2+^, Sr^2+^, Ba^2+^, Ra^2+^ and Pb^2+^ include gamma-ray counting uncertainties and the scatter in repeated experiments. The asymmetric error bars for No^2+^ represent the 68% confidence interval derived from Poisson counting statistics. The ionic radii of Ca^2+^, Sr^2+^, Ba^2+^, and Ra^2+^ are the values at CN = 6 (Pauling’s value), as used or derived in the literature by Silva *et al.* (*8*, *38*). The ionic radius of Pb^2+^ is the value at CN = 6, as reported by Shannon (*37*). The solid curve represents the quadratic reference trend fitted by weighted least-squares regression using only Ca^2+^, Sr^2+^, Ba^2+^, and Ra^2+^; Pb^2+^ and No^2+^ were excluded from the fitting.

### Interaction between No^2+^ and the crown ether

Since the unusually strong extraction of No^2+^ by the crown ether was not sufficiently explained by considering the ionic radius, it was necessary to investigate the electronic structure of the extracted chemical species in greater depth using relativistic quantum chemical calculations. The microstructure of the extracted No was deduced to be No(NO_3_)_2_(DtBuCH18C6), which is homologues to that formed in the extraction of Sr^2+^ by the HNO_3_/Sr resin system, as determined by EXAFS (*26*). The binding energies (BEs) of the M(NO_3_)_2_DtBuCH18C6 (M = Sr, Pb, and No) inclusion complexes were calculated by ADF program (*40*) along with the corresponding Wiberg bond indices, as listed in table S5. The obtained BEs suggested that the stability of the complexes decreased in the order No^2+^ > Pb^2+^ > Sr^2+^, which does not correspond with the order of their ionic radii (i.e., Pb^2+^ > Sr^2+^ > No^2+^), indicating that thermodynamic elemental selectivity is determined not only by the ionic radius, but also by the electronic state of the extracted complex. The higher bond indices for the Pb^2+^ and No^2+^ complexes are therefore considered to originate from covalent bonding. Additionally, the relative stability (ΔΔ*E*^ext^) values were calculated with respect to that of Sr, as shown in fig. S10. Notably, the ΔΔ*E*^ext^ values were in good agreement with the order of the experimental *K*_d_ values in 3.5 M HNO_3_, thereby accounting for the stronger extraction of No observed in the current experiments.

Table 1 lists the calculated natural charges for each fragment along with the natural electron configurations of the complex species determined by natural bond order analysis (ADF NBO program). The degree of charge transfer, Δ*Q,* was calculated by taking into account the difference between the initial charge (+2 for a divalent metal ion, 0 for the crown ether, and − 2 for the two NO_3_^−^ ions) and the *Q* value of the complex. It was deduced that the charges for Pb and No were clearly lower than those for Sr. Moreover, the charges of the crown ether O atom were comparable for all complexes, whereas the charges of the NO_3_^−^ O atoms in the Pb and No complexes are lower than the corresponding value for the Sr complex. This result indicates charge transfer from the NO_3_^−^ ions to the central metal ion in the Pb and No complexes. The results of energy decomposition analysis combined with natural orbitals for chemical valence (EDA–NOCV) analysis also support this charge transfer (see Supplementary Materials). From analysis of the natural electron configurations of the central metal atoms in the M(NO_3_)_2_DtBuCH18C6 complexes, it was found that the complex with No contained a contribution from 6d orbital, while the complex with Pb contained a contribution from the 6p orbital. The populations of the 7 s and 6d orbitals of No in No(NO_3_)_2_DtBuCH18C6 are identical to or slightly higher than those in [No(N_2_)_6_]^2+^ (*15*). Consequently, it was considered that covalent bonding contributed to the stability of the complex of Pb^2+^ and No^2+^, revealing the soft-acid character of No^2+^, with interactions between its 6*d* orbital and the ligand molecules.

**Table 1.** Natural charges (Q) and degrees of charge transfer (ΔQ) for the central metal ions, crown ether (L), and nitrate ions, along with the configurations of the central metal ions in the M(NO_3_)_2_DtBuCH18C6 complexes.

	Q(M)	Q(L)	Q(2NO_3_^−^)	ΔQ(M)	ΔQ(L)	ΔQ(2NO_3_^−^)	Configuration of the central metal ion
Sr	1.790	0.094	−1.884	−0.210	0.094	0.116	[core]5 s(0.13)4d(0.06)5p(0.01)
Pb	1.487	0.249	−1.735	−0.513	0.249	0.265	[core]6 s(1.98)6p(0.47)6d(0.01)7p(0.04)
No	1.312	0.352	−1.664	−0.688	0.352	0.336	[core]7 s(0.16)5f(13.99)6d(0.48)7p(0.01)

### Relativistic effect on the atomic orbitals of No^2+^

Because the fundamental objective of the chemistry of heavy elements is to probe the strong relativistic effects inherent to the heaviest elements, we further analyzed the bare ions using conceptual DFT for understanding the electron-accepting character of No^2+^. The absolute hardness *η* of an element is defined as (I−A)/2, where *I* is the ionization potential and *A* is the electron affinity (*41*–*43*). According to Koopman’s theorem (*44*), *I* and *A* can be calculated as $I=-E_{\text{HOMO}}$ and $A=-E_{\text{LUMO}}$, respectively. The calculated absolute hardness values are given in Fig. 3. As shown, the absolute hardness of No^2+^ calculated using the spin-orbit relativistic DFT approach was comparable to that of Pb^2+^. This result indicates that No^2+^ is a soft acid, unlike hard acids of Ca^2+^ and Sr^2+^. Interestingly, at the non-relativistic Hamiltonian level calculation, the value of the absolute hardness of No^2+^ is comparable to those of Sr^2+^ and Ba^2+^, indicating that relativistic effects render No^2+^ a soft acid. The atomic orbital of No^2+^ calculated using the non-relativistic and relativistic Hamiltonians [scalar or spin-orbit X2C approximation (*45*)] are compared in fig. S11. Notably, the relativistic effect destabilizes the 5*f* orbital as the HOMO and stabilizes 7 *s* orbital as the LUMO. As a result, the absolute hardness, which corresponds to the HOMO–LUMO gap, is reduced, and No^2+^ behaves as a soft acid. The strong extraction of No^2+^ by the crown ether therefore reflects the strong relativistic effect on the atomic orbital of No^2+^.

**Fig. 3.**
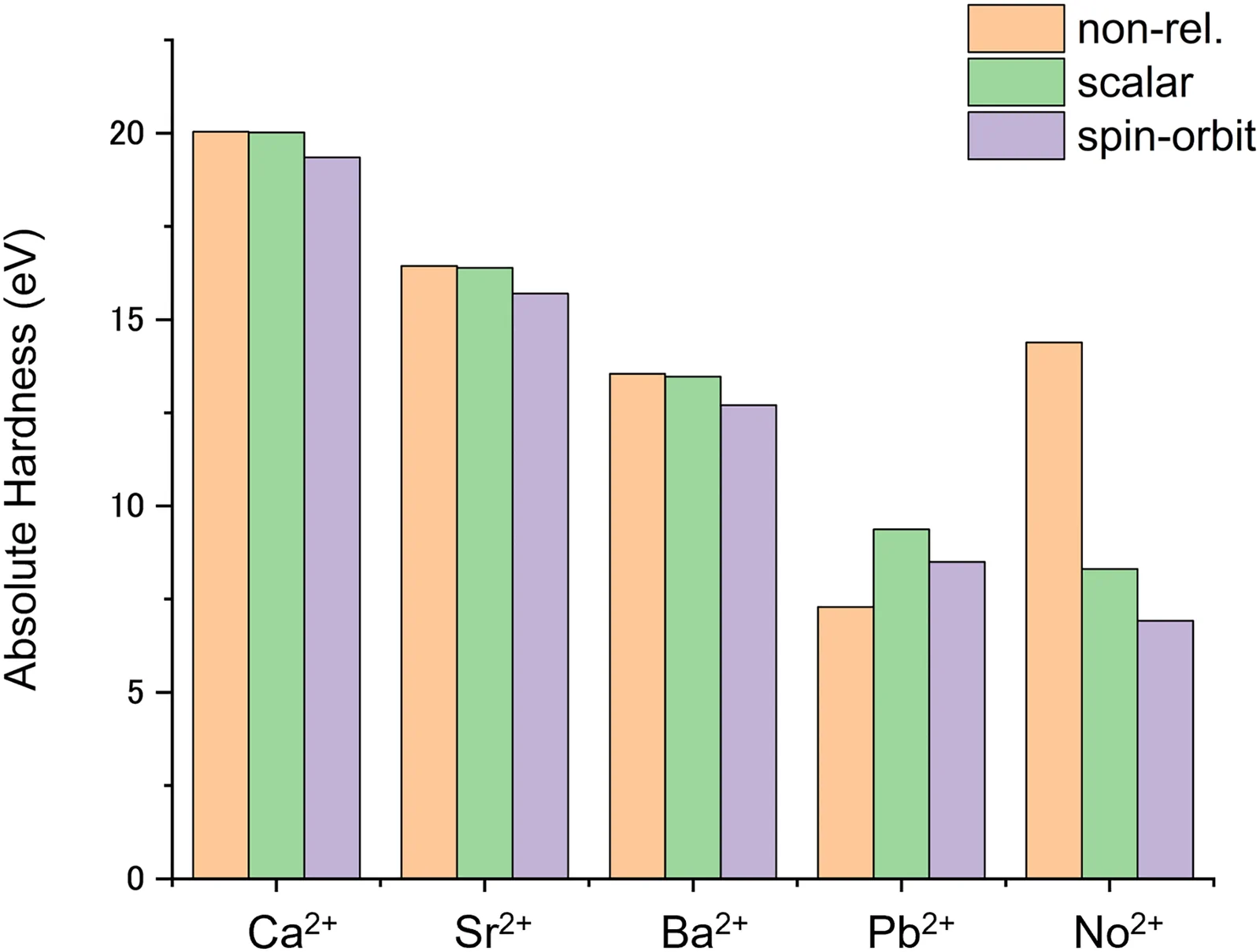
Calculated absolute hardness values for the divalent metal ions. All values are based on quantum chemical calculations for gas-phase (i.e. no solvation) conditions. The B3LYP functional and the STO-TZ2P basis set were used in ADF 2023 program (*40*). The values obtained using the non-relativistic Hamiltonian, spin-free x2c Hamiltonian, and exact two-component (x2c) relativistic Hamiltonian with spin-orbit coupling are represented by the orange (non-rel.), green (scalar), and purple (spin-orbit) bars, respectively.

## DISCUSSION

In this study, we successfully observed strong reactivity of No^2+^ with crown ethers and for the first time propose the softness of No^2+^ caused by strong relativistic effects. Specifically, it was demonstrated that using crown ethers, No^2+^ forms the extracted species with higher covalency than Ca^2+^ and Sr^2+^. These results clearly indicate that new aspects related to the fundamental chemical properties of such heaviest element could emerge upon examining their interactions with crown ethers (*31*, *32*, *46*).

The present results also indicate that the extraction behavior of No^2+^ should not be regarded as a simple extension of either alkaline-earth metal ions or Pb^2+^. Although Pb^2+^ is a useful reference for a softer divalent ion, the complex-level analyses and conceptual DFT for the bare ions suggest that No^2+^ has its own electronic character arising from relativistic effects. This difference can influence not only the stability of the extracted complex but also the extraction kinetics. Indeed, the apparent *K*_d_ value of No^2+^ increased from 60 s to 180 s, indicating that the latter represents a conservative estimate of the equilibrium extraction strength. Thus, even if extraction equilibrium is not reached within 180 s, the soft-acid interpretation of No^2+^ is not weakened; rather, this reinforces the assumption that No^2+^ has an electron-accepting character among divalent metal ions.

The sensitivity analysis of $|Z_{\mathrm{emp}}|$ also demonstrated that the inferred magnitude of the deviation of No^2+^ from the alkaline-earth reference trend depends on the assumed ionic radius. These results highlight that a more precise determination of the ionic radius of No^2+^ will be important for advancing to understand the chemical properties of nobelium.

## MATERIALS AND METHODS

### Offline experiments

To examine the time required to reach equilibrium in the extraction, the time dependences of the *K*_d_ values were obtained in the range of 60 s–24 h using solutions of 3.5 M HNO_3_ (^47^Ca, ^85^Sr, ^133^Ba, ^212^Pb, and ^226^Ra) and 7.6 M HCl (^47^Ca, ^133^Ba, and ^226^Ra). Hydrochloric acid and nitric acid (grade for analysis of poisonous metals; FUJIFILM Wako Pure Chemical Corporation, Japan) were used.

Sr resin (Eichrom, 26.2–360 mg) was mixed with 3.5 M HNO_3_, 7.6 M HCl or 5.3 M HClO_4_ (500 μL) solutions containing ^47^Ca, ^85^Sr, ^133^Ba, ^212^Pb, or ^226^Ra radiotracers in a 0.5 mL centrifugal PP filter units (Ultrafree-MC, Merck), and shaken for 1–1440 min using a vortex mixer at 25 ± 1°C. After the desired time, the liquid phase was subjected to centrifugation at 3000 rpm for 1 min, then only the liquid phase was filtered and weighed. Subsequently, the radioactivity of the solution sample was measured by counting the 1297-, 514-, 356-, 238-, or 186-keV γ-rays emitted from ^47^Ca, ^85^Sr, ^133^Ba, ^212^Pb, or ^226^Ra, respectively, using a Ge semiconductor detector. In all solid–liquid experiments, control experiments were performed without the resin to determine the radioactivity, *A*_STD_. (*33*) The *K*_d_ values mL g^−1^ were determined using Eq. (1)

$$K_{d}=\frac{\left(A_{\text{STD}}-A_{s}\right)V_{s}}{A_{s}w_{r}}$$ (1)

where *w*_r_ is the weight of the dry resin (g) and *V*s is the liquid phase volume (mL).

### Online experiments

The online experiment with AMBER was performed with ^255^No (*T*_1/2_ = 210 s) and ^162^Yb (*T*_1/2_ = 18.9 min) nuclides produced in the ^248^Cm(^12^C,5*n*)^255^No and ^nat^Gd(^12^C,*xn*)^162^Yb reactions, respectively, using the K 70 AVF cyclotron at RIKEN in the similar manner of that in the Ref (*5*, *47*). The 685.2 μg/cm^2^ of ^248^Cm and 69.6 μg/cm^2^ of ^nat^Gd mixture on the 1.880 mg/cm^2^ of Be backing foil was used as the target. The ^12^C beam was extracted with an energy of 89.9 MeV at typically ∼0.7 particle-μA. The nuclear reaction products, which recoiled from the target, were captured by KCl aerosols in He gas at ∼130 kPa. The products were transported to a chemistry laboratory by helium gas at a flow rate of 2.5 L/min. The radioactivity of ^162^Yb was monitored during a 1-d online repetitive extraction experiment.

For the purpose of the extraction experiment, the transported products were deposited on the collection site of an ASO slider (*33*) for 464 s. Subsequently, the deposited sample was dissolved in 3.5 M HNO_3_ or 7.6 M HCl (∼0.25 mL) for 15 s and then injected into a chemical reaction container containing 15.01–29.93 mg Sr resin. After mixing for 60 or 180 s, the solution phase was ejected from the container using compressed air (0.10–0.15 MPa) for 10 s and collected on a Ta disk placed on a turn-table. In the control experiment, the dissolved solution was shaken for 60 s without the Sr resin. The solution sample on the Ta disk was evaporated to dryness using a combination of hot He gas (1.4 L/min; 300°C and 0.6 L/min; 600°C) and a halogen heat lamp to prepare the dry sample for α-particle measurement. The Ta disk was then transferred to a detection chamber equipped with a Si PIN photodiode detector using a robot arm and the α-particle measurements were conducted (*48*). The eight detector chambers were used in turn. The experimental condition with 3.5 M HNO_3_ was chosen because the differences in *K*_d_ among Ca, Sr, Ba, and Ra were the largest, resulting in the highest element selectivity. The experimental condition with 7.6 M HCl was chosen because literature values for 8 M HCl are available and higher HCl concentrations are less affected by washing with 0.1 M HCl in repeated extraction experiments, making it possible to obtain stable extraction data.

Determination of the detection efficiency and energy calibrations were performed using ^241^Am (*E*_α_ = 5.49 MeV) and ^212^Bi (*E*_α_ = 6.07 MeV)–^212^Po (*E*_α_ = 8.78 MeV) decay series. The efficiencies and energy resolutions for the samples were ∼33% and ∼50 keV full width at half maximum at the 5.49-MeV α peak of ^241^Am, respectively. The α-particle measurements were conducted for ∼2400 s, beginning at ∼160 s after the start of product dissolution for the 60 s mixing case, and at ∼280 s for the 180 s mixing. After performing the α-particle measurements, the γ-ray activity of ^162^Yb (*E_γ_* = 163.35 keV) was measured using the Ge detector to determine the chemical yield. The chemical yield was determined by comparing the decay-corrected ^162^Yb activity recovered on the Ta disk after the AMBER extraction procedure with that initially collected on the glass filter. The ^162^Yb activity was determined from the 163.35-keV γ-ray peak measured with the Ge detector. The chemical yield was calculated using the formula

$$Y_{\text{chem}}=\frac{A_{\text{Ta}}}{A_{\text{GF}}}\cdot \frac{1}{1-f_{\text{ads}}}$$ (2)

where *A*_Ta_ is the decay-corrected activity of ^162^Yb on the dried Ta disk after the AMBER extraction procedure, and *A*_GF_ is the decay-corrected activity of ^162^Yb collected on the glass filter. The reference time was defined as the end of the dissolution process. The term $f_{\text{ads}}$ denotes the fraction of Yb adsorbed on the Sr resin during the extraction process. Thus, the factor $1/\left(1-f_{\text{ads}}\right)$ corrects the recovered solution-phase activity for the loss of Yb due to adsorption onto the resin.

The No and Yb species and all other nuclear reaction products adsorbed on the resin were stripped by washing the resin 5 times with of 0.1 M HCl (∼0.3 mL; 5 s mixing time). Subsequently, the resin was conditioned 2 times with HNO_3_ or HCl (∼0.16 mL; 5 s mixing time) for the subsequent extraction. All stripped and conditioned solutions were discarded. The Sr resin was reused 11–32 times prior to renewal. Control extraction experiments were also performed in the absence of Sr resin to evaluate *A*_r_.

The asymmetric error limits of the *K*_d_ values for No were evaluated based on the counting statics of the observed α-event ratios, using on the 68% confidence intervals for the Poisson distributed values (*49*).

### Analysis of ionic-size dependence

To evaluate the deviation of Pb^2+^ and No^2+^ from the extraction behavior expected from ionic size matching, the distribution coefficients were analyzed on a logarithmic scale

$$y={\text{log}}_{10}K_{d}$$ (3)

The size-based reference trend was fitted using only the alkaline-earth metal ions (Ca^2+^, Sr^2+^, Ba^2+^, and Ra^2+^). Pb^2+^ and No^2+^ were not included in the fitting. The reference trend was represented by

$$\hat{y}\left(r\right)=ar^{2}+\mathrm{br}+c$$ (4)

where *r* is the ionic radius and $\hat{y}\left(r\right)$ is the predicted value of ${\text{log}}_{10}K_{d}$.

As an empirical measure of the deviations of the reference data from the fitted trend, $\sigma_{\mathrm{emp}}$ was defined as the maximum absolute residual among the four reference ions

$$\sigma_{\mathrm{emp}}=\underset{i}{\text{max}}|y_{i}-\hat{y}\left(r\right)|$$ (5)

To quantify the deviation of Pb^2+^ and No^2+^ from the size-based reference trend, the standardized deviation, $Z_{\mathrm{emp}}$, was calculated as

$$z_{\mathrm{emp}}\left(r\right)=\frac{{\text{log}}_{10}K_{d}-\hat{y}\left(r\right)}{\sigma_{\mathrm{emp}}}$$ (6)

A positive $Z_{\mathrm{emp}}$ means that the observed *K*_d_ value is higher than that expected from the size-based trend, whereas a negative value implies that the *K*_d_ value is lower than the expected value.

To evaluate the sensitivity to the uncertainty in the ionic radius of No^2+^, $Z_{\mathrm{emp}}$ was calculated over the reported range of

$$1.00\;Å\leq r_{\text{No}}\leq 1.10\;Å$$ (7)

with 1.05 Å used as the representative value.

ChatGPT using GPT-5.5 Thinking (OpenAI) was used as an auxiliary tool for drafting Python scripts and checking the implementation of the statistical analysis workflow. The final analysis, validation of the code, and interpretation of the results were performed by the authors.

### Theoretical calculations

All calculations reported herein were performed using the ADF package (*40*), the DFT approach was used to calculate the extraction system. For the molecular calculations, geometry optimization was performed using DZP basis sets for all atoms along with the B3LYP functional at the scalar-ZORA level. For each optimized structure and single M^2+^ ion, more accurate DFT calculations (single-point calculations) were performed using TZ2P basis sets for all atoms in combination with the B3LYP functional at the spin-orbit ZORA level. Geometrical optimization was performed without solvent effects, while single-point calculations were carried out both with and without solvent effects. To consider the solvent effects for calculation of the ΔΔ*E*^ext^ value, the SM12 solvation model was employed (*50*). The binding energy (BE) in the gas phase was calculated using the energy of each fragment in the gas phase

$$\text{BE}=E_{M{\left({\text{NO}}_{3}\right)}_{2}L}-\left(E_{M}+E_{L}+2E_{{\text{NO}}_{3}}\right)$$ (8)

The extraction energy with solvent effects, Δ*E*^ext^, was calculated based on the following extraction reaction

$$\begin{array}{c} {\left[M{\left(H_{2}O\right)}_{n}\right]}_{\text{aq}}^{2+}+L_{\text{octanol}}+2{{\text{NO}}_{3}^{-}}_{\text{aq}}\to \text{ML}{{\left({\text{NO}}_{3}\right)}_{2}}_{\text{octanol}}\\ +nH_{2}O_{\text{aq}},\;\left(n=8\;\text{for}\;\text{Ca},\text{Sr},\text{Ba},\text{Ra},\text{and}\;\text{No},n=7\;\text{for}\;\text{Pb}\right) \end{array}$$ (9)

With regard to the CN of the hydrated No^2+^ ion, the calculated value of CN = 8 was adopted based on ab initio molecular dynamics simulations employing a relativistic effective core potential (*10*).

The reference values used to benchmark the conceptual DFT calculations for monatomic ions were derived from the ionization energies reported in the NIST Atomic Spectra Database (*51*).
