# Supplementary material for: 102No2+ as a soft metal ion: Extraction of 255No using crown ethers
Source: Sci Adv. 2026 Sep 30;12(40):eaeh4903. doi: 10.1126/sciadv.aeh4903 (PMC13626048; doi:10.1126/sciadv.aeh4903)
Supplement: Supplementary file 1 [file sciadv.aeh4903_sm.pdf]

Science Advances
AAAS

Supplementary Materials for

$_{102}No^{2+}$ as a soft metal ion: Extraction of ^{255}No using crown ethers

Eisuke Watanabe *et al.*

Corresponding author: Eisuke Watanabe, ewata@post.kek.jp; Yoshitaka Kasamatsu, kasa@chem.sci.osaka-u.ac.jp

Sci. Adv. **12**, eaeh4903 (2026)
DOI: 10.1126/sciadv.aeh4903

This PDF file includes:

Supplementary Text
Figs. S1 to S12
Tables S1 to S8

Supplementary Text

Mock-up experiment using AMBER

In the offline experiment using AMBER as a mock-up experiment for ^{255}No, the solid–liquid extraction of ^{133}Ba (a representative element of alkaline earth metal) was performed by the following procedure. The 3.2–47.6 mg of the Sr resin and 300–500 µL of 3.5 M HNO_3, 7.6 M HCl or 5.3 M $HClO_4$ containing the ^{133}Ba were manually put in a reaction container in AMBER and mixed at ~20 ºC for 60 s using the vortex mixer. Subsequently, only the solution phase was pushed out of the container by the compressed air and was collected in a PP tube. The ^{133}Ba adsorbed on the resin were washed by 9 times of rinse with ~0.3 mL of stripping solution (0.1 M HCl or 0.01 M HNO_3) for 5 s. Each solution phase was pushed out of the container and was collected in the PP tubes. All the solution samples were subjected to γ-ray measurement using a Ge detector. For indirect measure of the radioactivity on the resin by the method described in the batch extraction experiment, a control extraction experiment without the resin was also performed.

In this study, the K_d values were calculated by two methods (*33*). The first is a control method using a standard solution according to Eq. 1 in the main text. The second is the 2-solution method, which uses the radioactivity of the stripped solution. In the 2-solution method, the following equation was used,

$$K_{\mathrm{d}} = \frac{\left(\sum_{i=1}^{n} A_i^{2nd}\right) V_s}{A_S w_r}, \qquad \text{Eq. (S1)}$$

where A^{2nd}_k is radioactivity of the k-th stripped solution and n is the total number of back extraction (n = 9 in this study).

The recovery rate until m-th back-extraction, $R(m)$, was defined by the following equation:

$$R(m) = \frac{\sum_{i=1}^{m} A_i^{2nd}}{\sum_{i=1}^{n} A_i^{2nd}} \times 100, \qquad \text{Eq. (S2)}$$

R equals 100 (%) when Ba is stripped completely from the Sr resin.

Table S1 lists the obtained K_d values of ^{133}Ba in 3.5 M HNO_3, 7.6 M HCl, and 5.3 M $HClO_4$, using AMBER. The K_d values using AMBER agree with those from the batch experiment. Besides, the K_d values evaluated by the control method are consistent with those calculated by the 2-solution method, suggesting that the K_d values in the solid–liquid extraction in the present reaction system can be obtained by both methods using AMBER.

Figure S1 shows $R(m)$ in the back-extraction of after solid–liquid extraction in 3.5 M HNO_3 as a function of m, using 0.01 M HNO_3 and 0.1 M HCl as the back-extraction reagent. This graph shows that about 6 times back extractions are required to wash out all of the ^{133}Ba adsorbed on the resin in 0.01 M HNO_3, while 4 times extraction is enough in 0.1 M HCl. It is concluded that 0.1 M HCl can recover adsorbed metal ion faster than 0.01 MHNO_3.

Results of online solid–liquid extraction of ^{255}No

Table S2 summarizes the results of the online experiment of ^{255}No in RIKEN. Fig. S2 plots alpha-particle spectra obtained in the experiments. Figure S3 plots the K_d values of No on the mixing time in the system of Sr resin with 3.5 M HNO_3. In Figure S4, the K_d values of Ca, Sr, Ba, Ra, and No with the Sr resin in HCl were plotted.

Results of analysis for deviation from the size-based reference trend

Fig. S5 shows the results of the deviation band analysis for the size-based reference trend. Pb^{2+} and No^{2+} are located on the high-K_d side of the alkaline-earth reference trend, indicating that their extraction behavior is stronger than that expected from ionic size alone. Fig. S6 shows the dependence of $|Z_{emp}|$ on the possible ionic radius of No^{2+}. At the representative value of 1.05Å, the lower bound of the propagated 68% range of $|Z_{emp}|$ remained greater than 3. This result suggests that the experimental value for No^{2+} lay more than $3\sigma_{\mathrm{emp}}$ above the reference trend even when the measurement uncertainty was taken into account. Near the upper end of the examined ionic-radius range, the lower bound of the 68% range of $|Z_{emp}|$ fell below 1, indicating that a deviation within $1\sigma_{emp}$ could not be excluded when a larger ionic radius was assumed. Thus, although the magnitude of the inferred deviation depended on the assumed ionic radius of No^{2+}, the upward deviation exceeding $3\sigma_{emp}$ at the representative value of 1.05 Å was retained after accounting for the asymmetric 68% measurement interval of No^{2+}. For Pb^{2+}, the propagated 68% confidence interval of $|Z_{emp}|$ was 1.973–2.197.

Validation of coordination mode of NO_3^- for $No^{2+}$by geometry optimization calculation with DFT

To investigate the coordination modes of nitrate ions with No^{2+} in the extracted species, geometry optimization calculations were performed for $M(NO_3^{\mathrm{bidentate}})_2$DtBuCH18C6 or $M(NO_3^{\mathrm{bidentate}})(NO_3^{\mathrm{monodentate}})$ DtBuCH18C6, and the total energy was obtained as E_{bb} or E_{bm}, respectively. Relativistic ZORA-DFT calculations at the BP86/TZVP level under CPCM (water) solvation were performed by ORCA ver. 6.1. Table S3 lists the ΔE values ($\Delta E_{\mathrm{bidentate}} = E_{\mathrm{bm}} - E_{\mathrm{bb}}$). Figure S7 shows the structure of $M(NO_3)_2$DtBuCH18C6 used for input information of theoretical calculation. The stability of the bidentate ligand was demonstrated, regardless of the type of metal ion. These results are consistent with the experimental observations of bidentate ligands in Sr and Pb (*26*, *39*). Based on these results, the coordination model of nitrate with NO_2^+ was inferred to be $N_O(NO_3^{\mathrm{bidentate}})_2$DtBuCH18C6.

Optimized structure of $M(NO_3)_2$DtBuCH18C6 obtained by theoretical calculation

The structure of $M(NO_3^{\mathrm{bidentate}})_2$DtBuCH18C6, shown in (a) of Fig. S7, was used as the input information for theoretical calculations using the ADF program. The interatomic distances in optimized structures of $M(NO_3)_2$DtBuCH18C6 (M = Sr, Pb, and No) are listed in Table S4. The distances of C, N, and O from central metal follows the order of Pb > Sr > No, and this sequence is consistent with the ionic radius metal ion ($Pb^{2+} > Sr^{2+} > No^{2+}$).

EDA-NOCV analysis of $M(NO_3)_2$DtBuCH18C6

Table S6 lists the calculated total binding energy (TBE), orbital interaction (E_{orb}), and electrostatic interaction ($E_{lstatic}$) [kcal mol^{-1}] between M and $(NO_3)_2$DtBuCH18C6 (M = Sr, Pb, or No) based on EDA analysis. The E_{orb} values show the trend Sr < No < Pb, while the $E_{lstaatic}$ values follow the trend Sr < Pb < No. Table S7 lists the calculated NOCV energy [kcal mol^{-1}], its contribution to electrostatic interaction [%], and the degree of electron transfer from the ligand to the central metal ion [*ν*] in $M(NO_3)_2$DtBuCH18C6 (M = Sr, Pb, or No). The electron mobility of representative NOCVs is slightly higher in the No^{2+} complex than in the Sr^{2+} complex. Fig. S12 shows the isodensity surfaces (0.0009 me a.u.$^{-3}$) for the deformation maps relative to the top three contributions of $M(NO_3)_2$DtBuCH18C6 (M = Sr, Pb, or No).

Comparison of absolute hardness value by several quantum chemical methods

Benchmark calculations were performed to verify the validity of the absolute hardness calculations for various divalent bare ions (Ca^{2+}, Sr^{2+}, Ba^{2+}, Ra^{2+}, Pb^{2+}, Yb^{2+}, and No^{2+}). MP2 calculations and DFT calculations with the B3LYP, BHandHLYP, and BP86 functionals were performed using STO-TZ2P basis sets by ADF 2023. The simulation was performed at the exact two-component (x2c) relativistic Hamiltonian level with the spin-orbit coupling effect. Among the tested methods, the B3LYP functional gave the smallest RMSD to the reference values as listed Table S8. Therefore, B3LYP was adopted for the main conceptual DFT analysis of the bare ions.

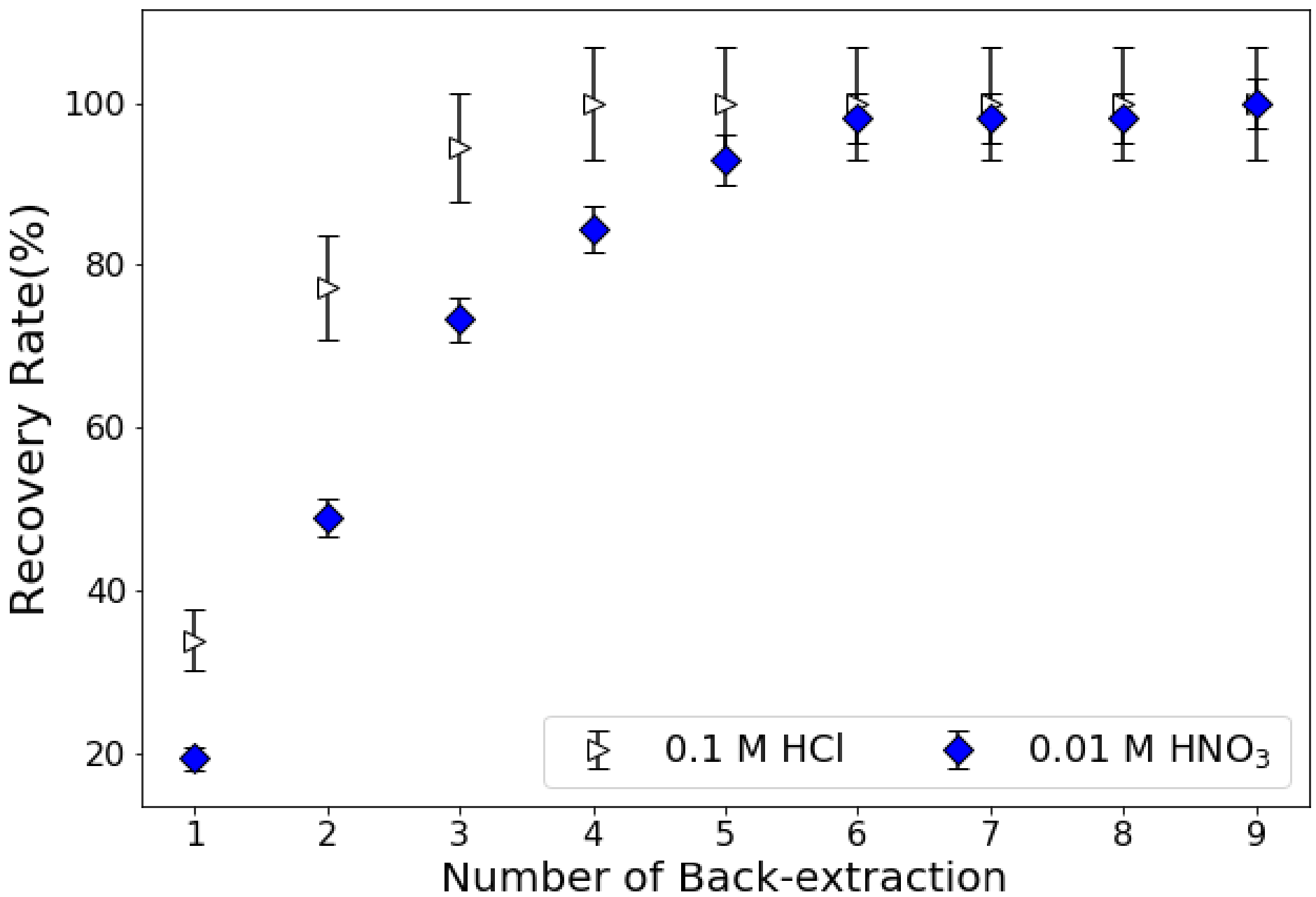

Fig. S1. The recovery rate of ^{133}Ba as a function of number of back extractions from 0.1 M HCl and 0.01 M HNO_3 after solid–liquid extraction experiment with the Sr resin in 3.5 M HNO_3.

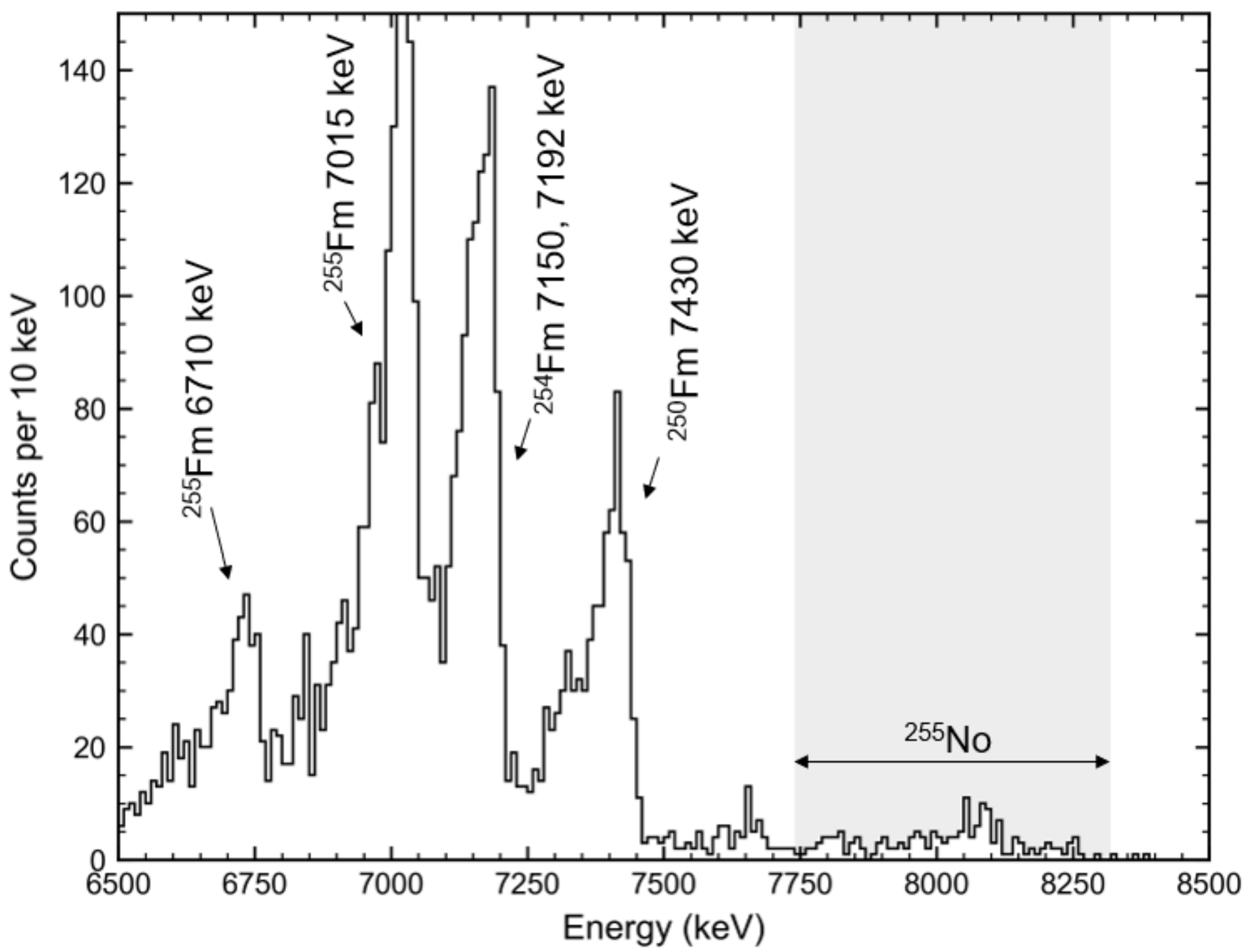

Fig. S2. α-particle spectra obtained in the online experiment. The α events obtained under all the conditions shown in Table S2 have been combined.

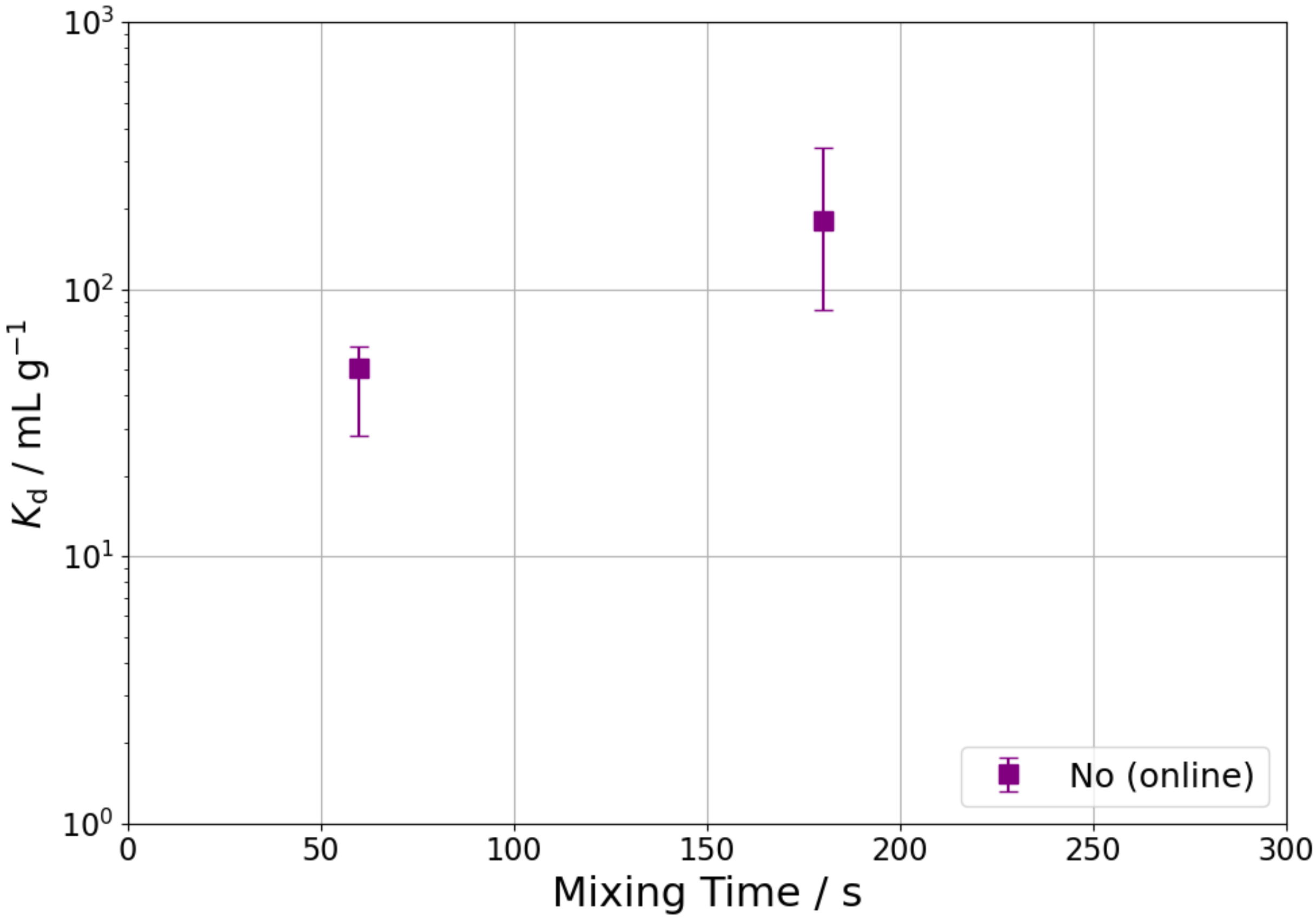

Fig. S3. K_d **values of** ^{255}**No on the mixing time in the system of Sr resin with 3.5 M** HNO_3**.**

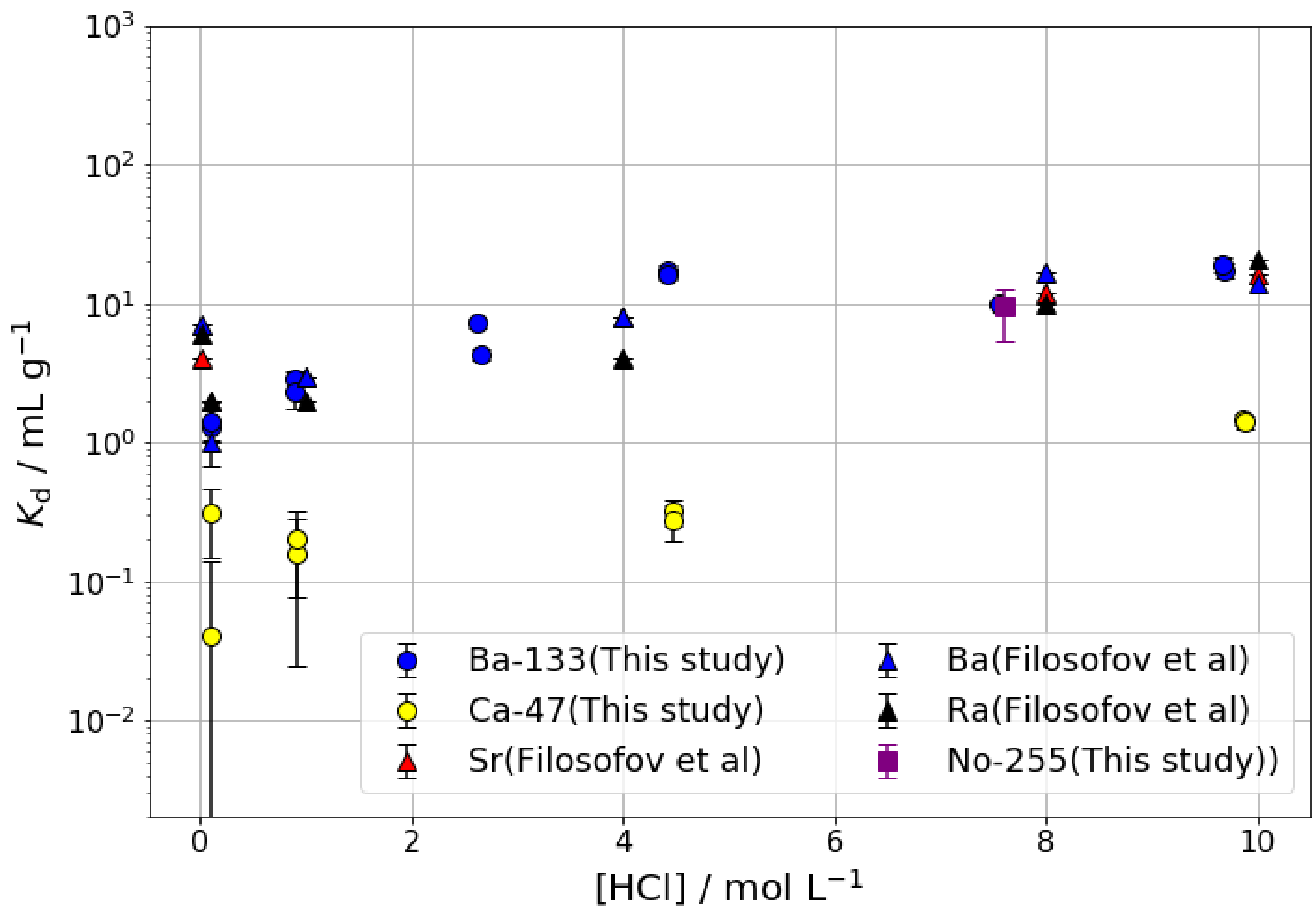

Fig. S4. K_d values of Ca, Sr, Ba, Ra, and No with the Sr resin in HCl.

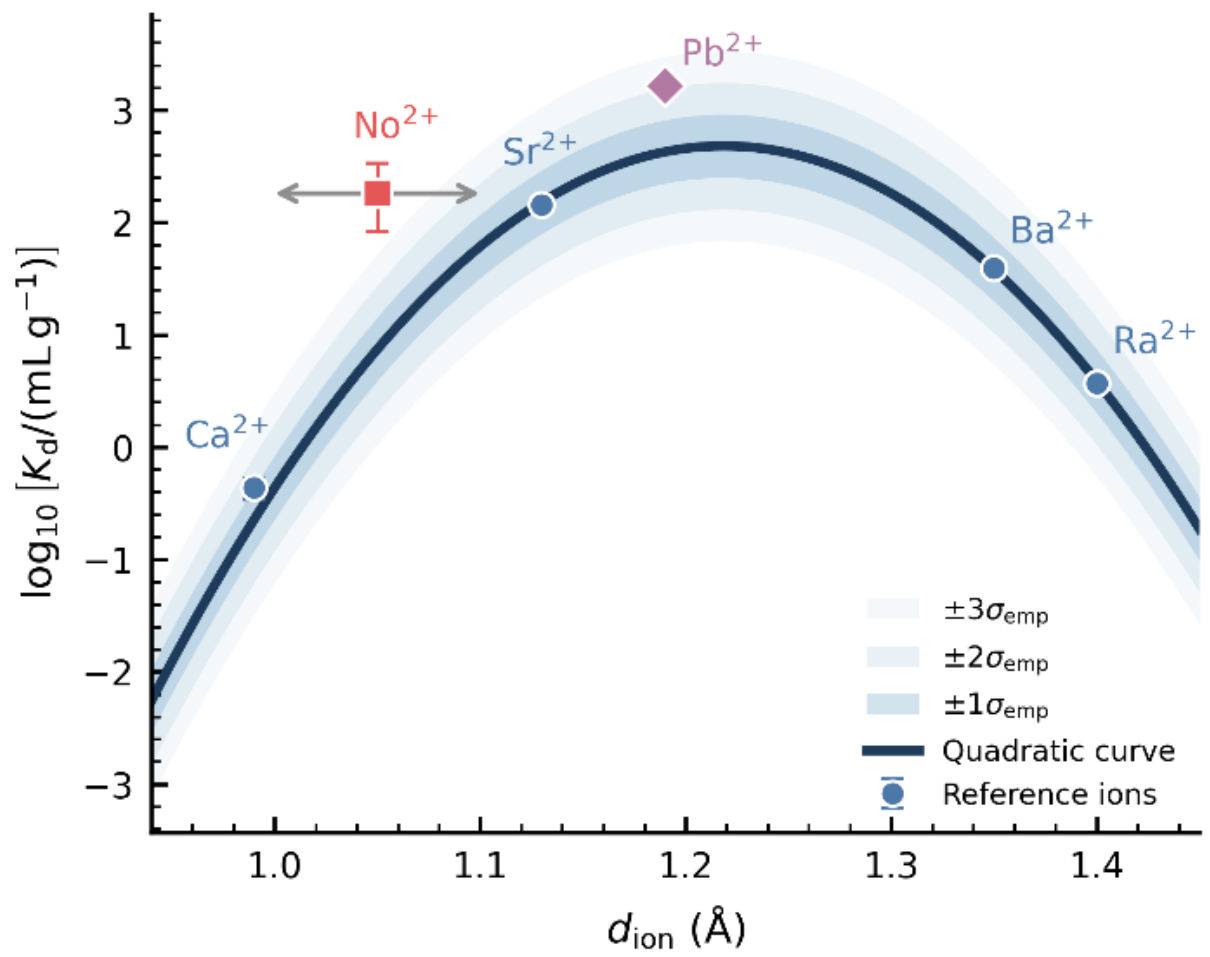

Fig. S5. Deviation band analysis of the size-based reference trend. The size-based reference trend was fitted from the K_d values of Ca^{2+}, Sr^{2+}, Ba^{2+}, and Ra^{2+}. The solid curve represents the fitted reference trend, and the shaded region represents the deviation within $1\sigma_{emp}$, $2\sigma_{emp}$, and $3\sigma_{emp}$ of the fitted trend, where σ_{emp} was defined as the maximum residual among the four reference ions. Pb^{2+} and No^{2+} were excluded from the fitting.

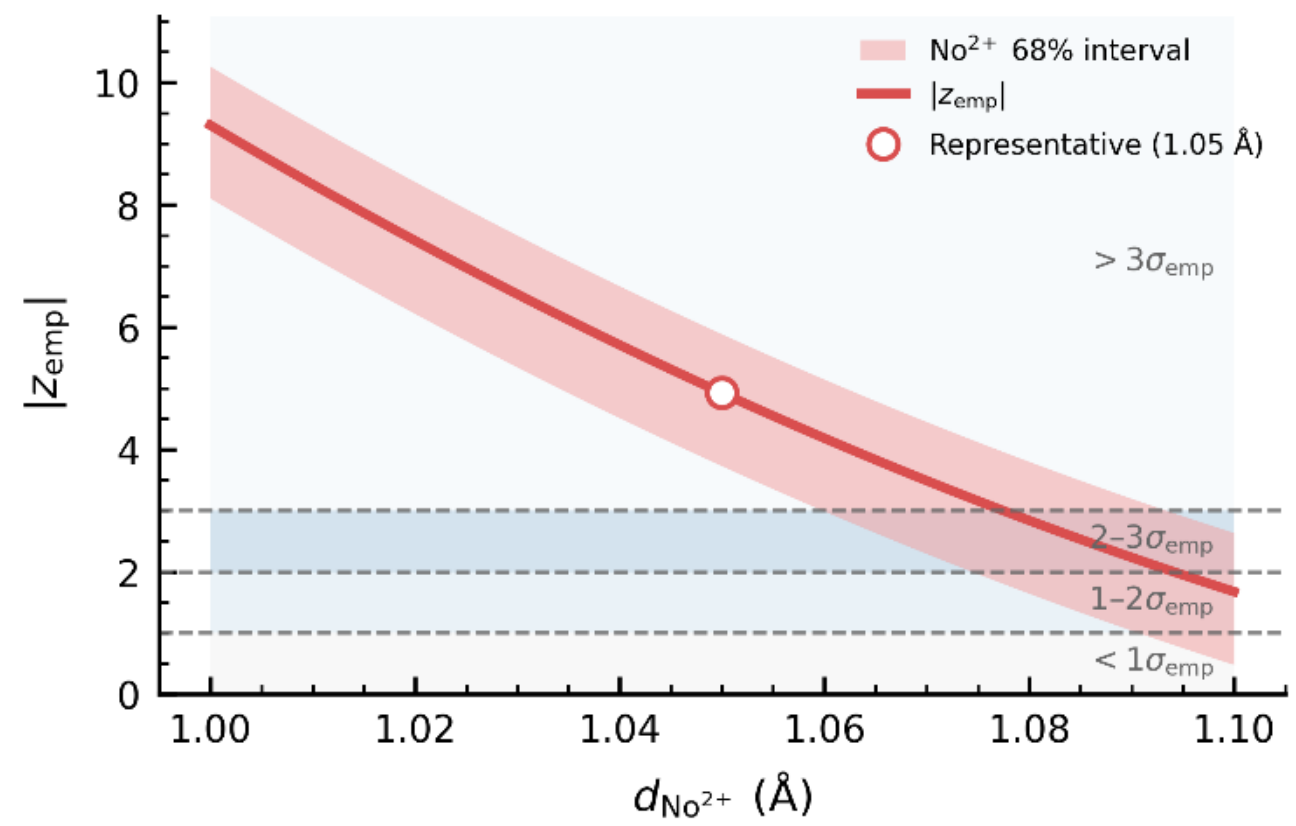

Fig. S6. Dependence of $|\boldsymbol{Z}_{\mathbf{emp}}|$ on the assumed ionic-radius range of $\mathbf{No^{2+}}$. The $|\boldsymbol{Z}_{\mathbf{emp}}|$ value is plotted over the reported ionic-radius range of No^{2+}. The solid red curve represents the value calculated from the nominal K_d, and the red shaded region represents the range propagated from the asymmetric 68% measurement interval of the No^{2+}. The open circle indicates the representative ionic radius of 1.05 Å. The background regions denote deviations of $< 1\sigma_{emp}$, $1\text{–}2\sigma_{emp}$, $2\text{–}3\sigma_{emp}$, and $> 3\sigma_{emp}$.

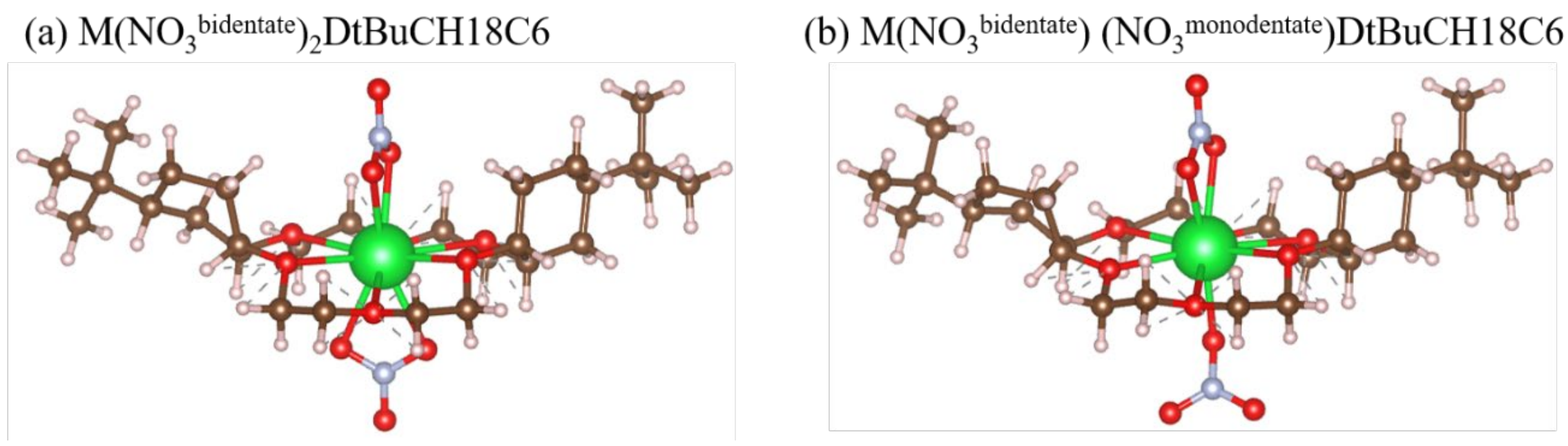

Fig. S7. Representative structures of $M(NO_3)_2$DtBuCH18C6 with two bidentate nitrate ligands (a) and one monodentate and one bidentate nitrate ligand (b).

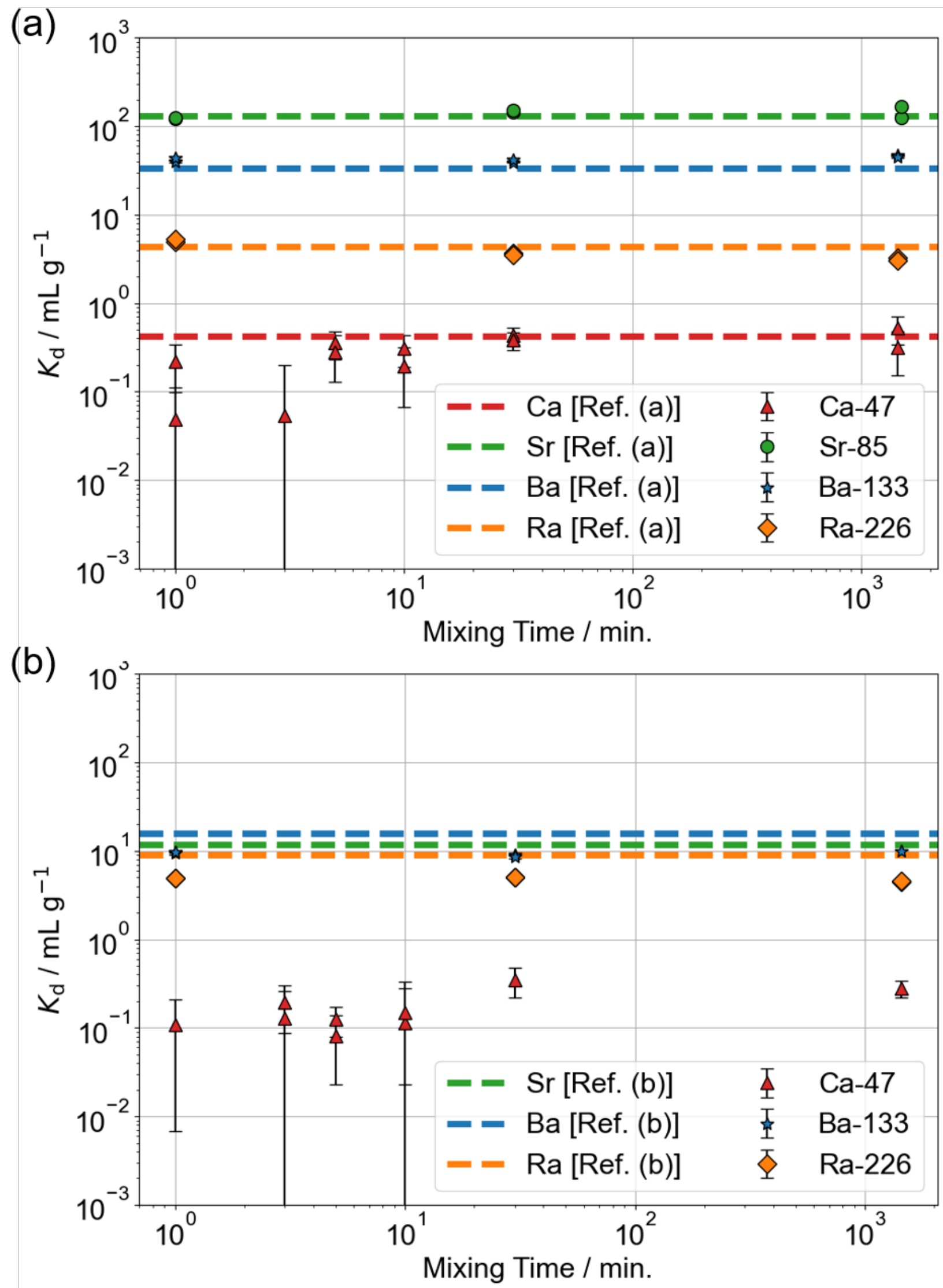

Fig. S8. K_d values of Ca, Sr, Ba, and Ra with different mixing times in (a) 3.5 M HNO_3 and (b) 7.6 M HCl solutions in the extraction performed using the Sr resin. The literature values provided in the figure are taken from the previous studies (*24*) or (*27*) as Ref (a) or (b), respectively.

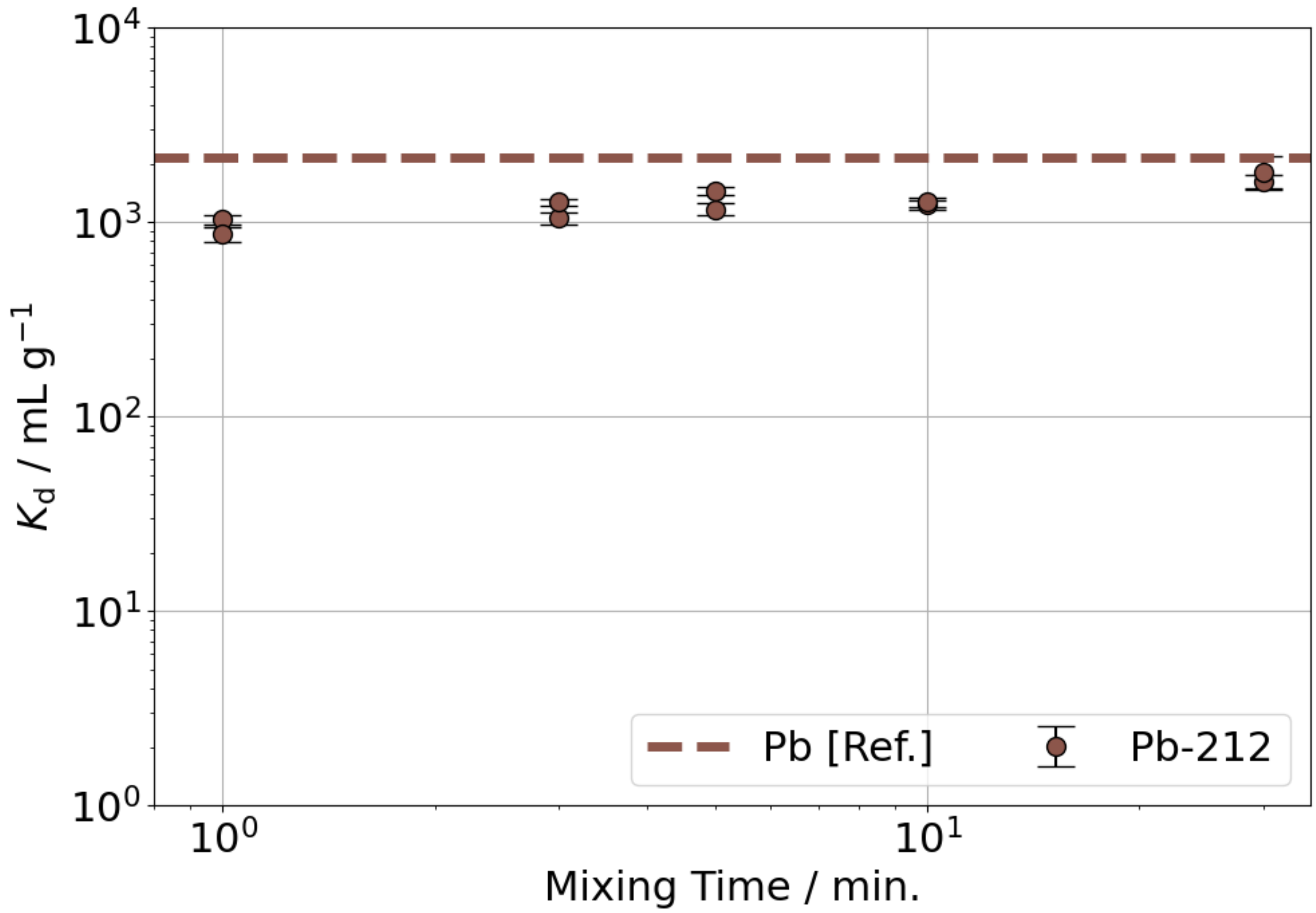

Fig. S9. K_d values of Pb for different mixing time in a 3.5 M HNO_3 solution in the extraction performed using the Sr resin. The literature value provided in the figure is taken from a previous study (*24*) .

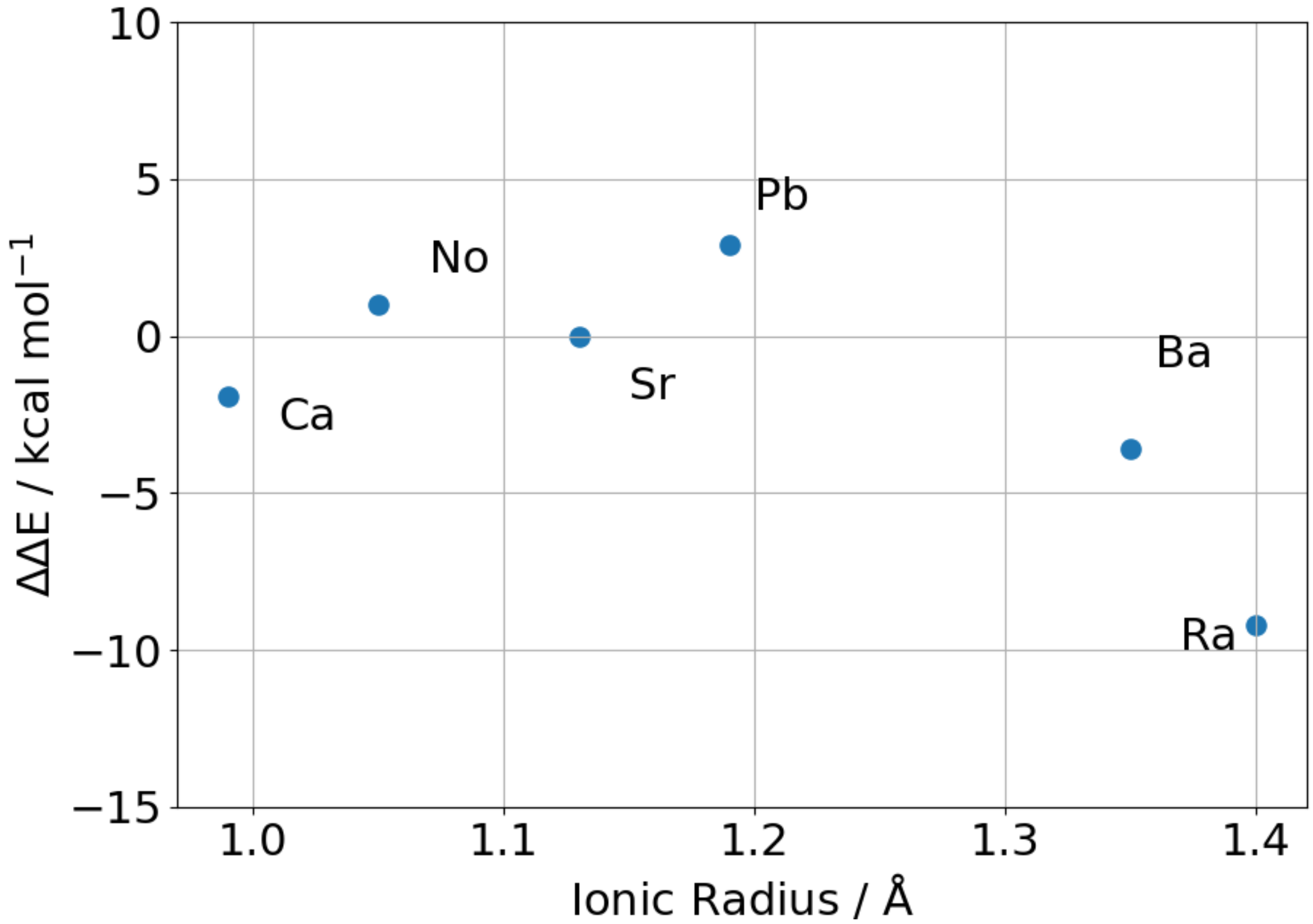

Fig. S10. Calculated $\Delta\Delta E^{ext}$ values as a function of the ionic radius of M^{2+}

.

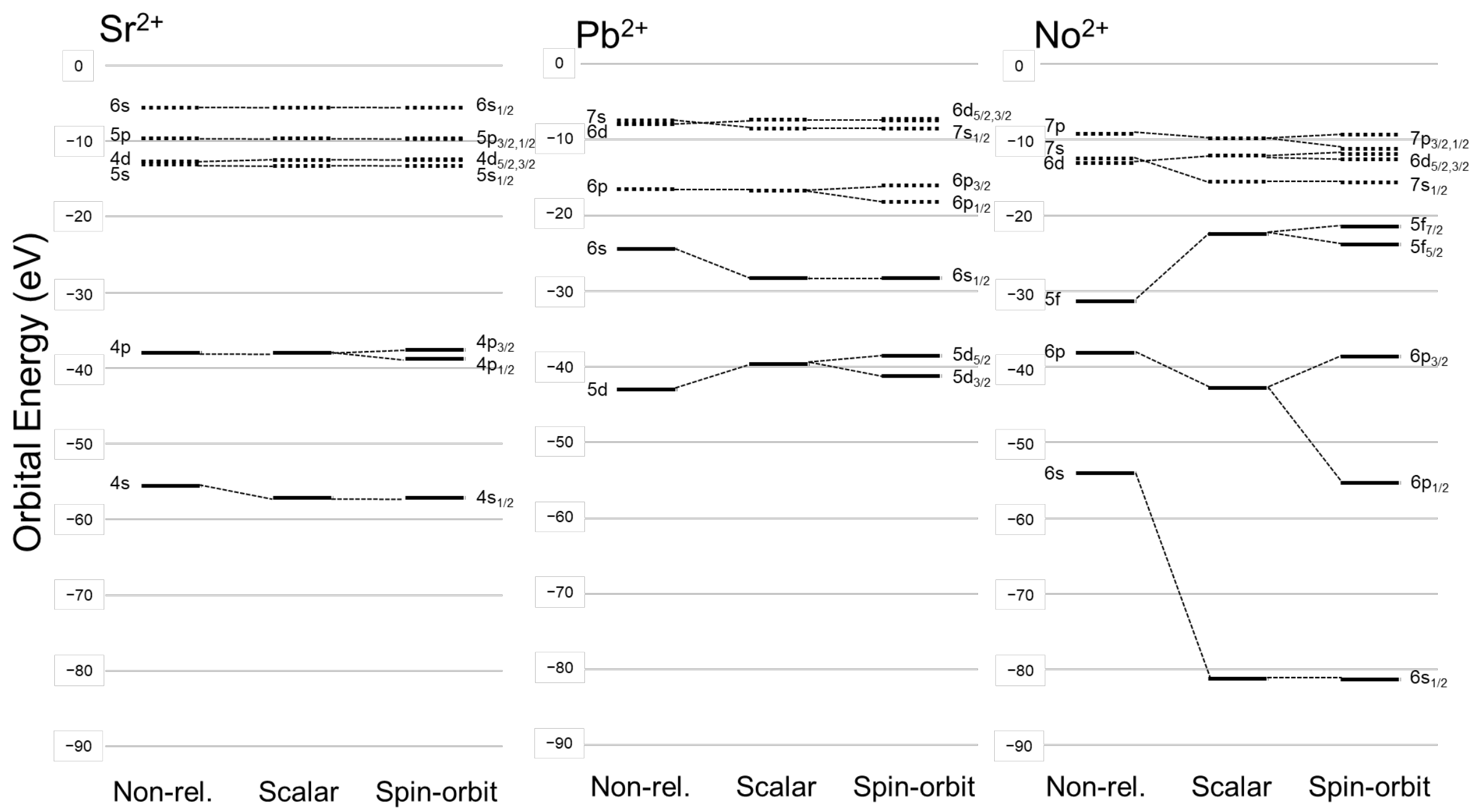

Fig. S11. The orbital energies of Sr^{2+}, Pb^{2+}, and No^{2+} were calculated using DFT calculations at the non-relativistic level, scalar relativistic level, and spin-orbit relativistic levels. Solid lines represent occupied electron orbitals, while dashed lines represent unoccupied orbitals.

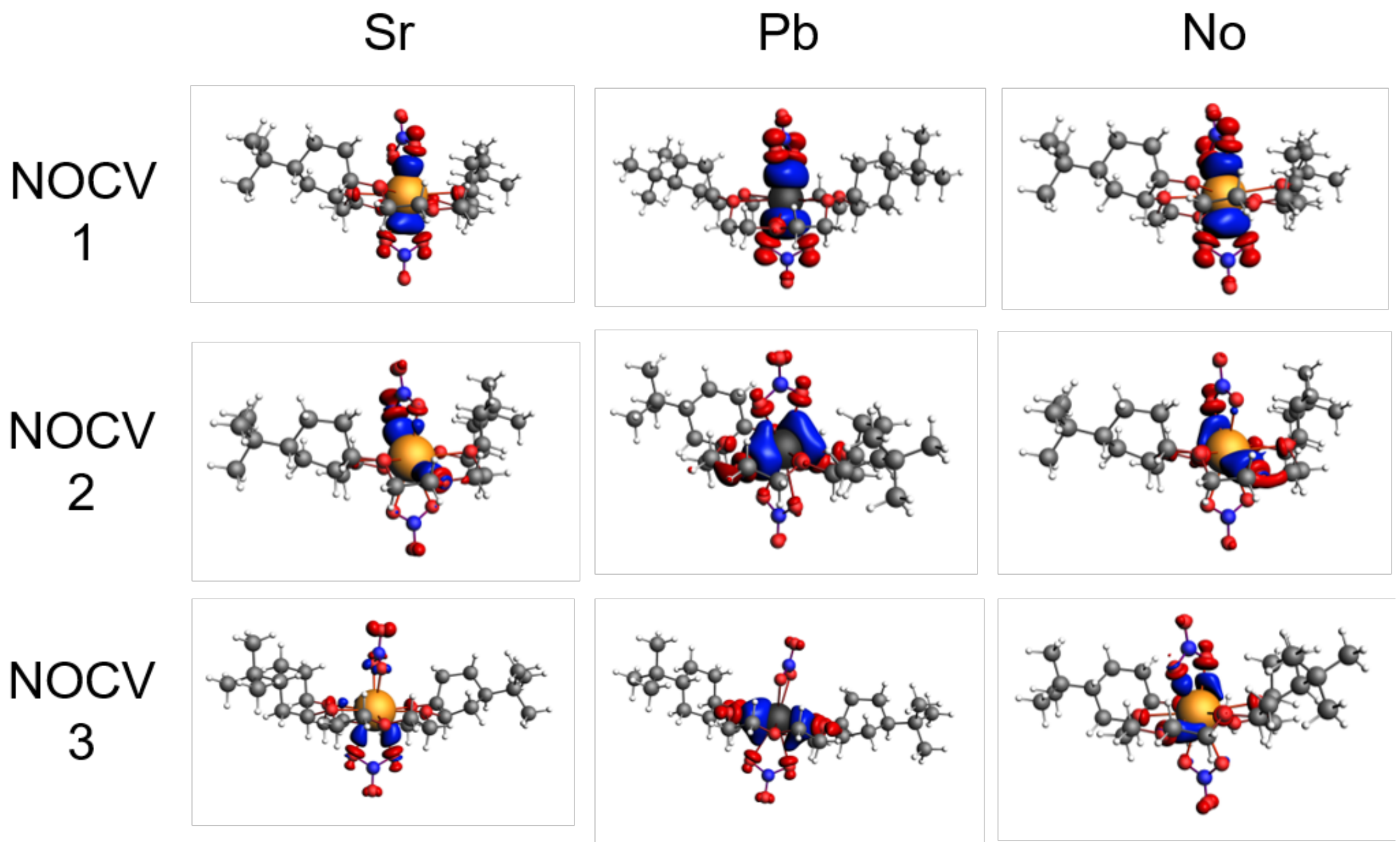

Fig. S12. Isodensity surfaces (0.0009 me a.u.$^{-3}$) for the deformation maps relative to the top three contributions of M(NO_3)$_2$DtBuCH18C6 (M = Sr, Pb, or No). The charge flux is red to blue.

Table S1. Distribution coefficients of ^{133}Ba with the Sr resin in 3.5 M HNO_3, 7.6 M HCl, and 5.4 M $HClO_4$ [mL g^{-1}].

	3.5 M HNO_3			7.6 M HCl			5.3 M $HClO_4$		
AMBER (2-soln)	45.4	±	2.6	9.4	±	0.6	1109.6	±	57.7
AMBER (control)	54.5	±	3.3	10.8	±	1.1	1315.2	±	58.7
Batch	47.1	±	1.2	9.7	±	0.5	1200.0*		

*reference (*27*)

Table S2. Summary of online experiment of ^{255}No.

	Aqueous phase	Mixing time /s	Beam Dose /10^{16}	Number of cycles	Chemical yield /%	α-count	K_d/mL g^{-1}
Control experiment	3.5 M HNO_3	60	4.22	15	22.1 - 60.5	79	-
	7.6 M HCl	60	2.72	9	20.7 - 39.6	49	-
Solid–liquid extraction	3.5 M HNO_3	180	6.67	19	19.7 - 86.2	5	181^{+157}_{-98}
	3.5 M HNO_3	60	3.55	16	28.7 - 52.8	23	50^{+10}_{-22}
	7.6 M HCl	60	3.23	13	11.9 - 23.5	17	7^{+10}_{-2}

Table S3. Calculated $\Delta E_{bidentate}$ values.

	$\Delta E_{bidentate}$
Sr	+13.2 kJ/mol
Pb	+8.5 kJ/mol
No	+12.8 kJ/mol

Table S4. Interatomic distance in $M(NO_3)_2$DtBuCH18C6 (M = Sr, Pb, and No) [Å]. O^{crown}, $O^{NO3, inner}$, and $O^{NO3, outer}$ indicate O atom of DtBuCH18C6, O atom directly coordinated to M of NO_3 ligand, and O atom non-coordinated to M of NO_3 ligand, respectively.

	M–O^{1st}	M–O^{crown}	M–$O^{NO3, inner}$	M–$O^{NO3, outer}$	M–N	M–C^{crown}
Sr	2.709	2.762	2.630	4.287	3.061	3.603
Pb	2.758	2.810	2.680	4.335	3.110	3.621
No	2.673	2.741	2.569	4.222	2.997	3.586

Table S5. Calculated Binding energies (BE) [kcal mol^{-1}] and Bond indices (BI) in M(NO$_3$)$_2$DtBuCH18C6 (M = Sr, Pb, or No).

	BE	BI (M^{2+})	BI (O_{crown})	BI (O_{inner})	BI (O_{outer})
Sr	−487.2	0.403	2.015	1.780	1.996
Pb	−498.5	1.043	2.053	1.839	2.003
No	−516.1	1.334	2.076	1.866	2.004

Table S6. Calculated total binding energy (TBE), orbital interaction (E_{orb}), and electrostatic interaction ($E_{lstatic}$) [kcal mol^{-1}] between M and $(NO_3)_2$DtBuCH18C6 (M = Sr, Pb, or No) based on EDA analysis.

	TBE	E_{orb}	E_{lstat}
Sr	-572.48	-151.2	-503.14
Pb	-575.31	-171.63	-510.56
No	-596.74	-167.02	-531.58

Table S7. Calculated NOCV energy [kcal mol^{-1}] , its contribution to electrostatic interaction [%], and degree of electron transfer from ligand to central metal ion [ν] in $M(NO_3)_2$DtBuCH18C6 (M = Sr, Pb, or No).

	Sr			Pb			No		
NOCV No.	Energy (kcal/mol)	Contribution (%)	ν[a)]	Energy (kcal/mol)	Contribution (%)	ν[a)]	Energy (kcal/mol)	Contribution %	ν[a)]
1	-13.926	9.210	0.100	-26.086	15.199	0.215	-16.230	9.717	0.124
2	-11.004	7.278	0.074	-21.982	12.808	0.143	-13.183	7.893	0.060
3	-9.484	6.272	0.057	-20.810	12.125	0.141	-12.174	7.289	0.042
4	-9.818	6.493	0.037	-6.255	3.644	0.015	-10.465	6.266	0.065
5	-9.023	5.968	0.045	-5.279	3.076	-	-10.676	6.392	0.045
6	-7.846	5.189	0.023	-4.880	2.843	-	-9.649	5.777	0.048
7	-4.995	3.304	0.013	-5.214	3.038	-	-5.212	3.121	-
8	-5.052	3.342	-				-5.182	3.103	-
9	-4.842	3.202	-				-5.021	3.006	-
10	-5.010	3.314	-						
Total	-81.00	53.57		-90.50	52.73		-87.79	52.56	

a): ν values of 0.01 or less are denoted by "-".

Table S8. Absolute hardness (η) in eV unit of M^{2+} with various methods. Reference values are calculated from the NIST database (*51*).

M^{2+}	MP2	BP86	B3LYP	BHandHLYP	Ref.
Ca^{2+}	19.41	19.40	19.35	19.40	19.5207(3)
Sr^{2+}	15.82	15.73	15.70	15.80	15.9266(2)
Ba^{2+}	12.84	12.72	12.71	12.82	12.920(3)
Yb^{2+}	7.01	6.68	6.42	5.79	6.44(3)
Pb^{2+}	8.56	8.60	8.50	8.25	8.452(1)
No^{2+}	6.71	7.05	6.92	6.60	6.74(40)
RMSD	0.25	0.21	0.16	0.29	
